# Long-term use of tDCS–clinical and ethical considerations

**DOI:** 10.3389/fnhum.2026.1792050

**Published:** 2026-04-24

**Authors:** Elodie Malbois, Samia Hurst, Raffaele Rodogno, Adrian G. Guggisberg

**Affiliations:** 1 Institute for Ethics, History, and the Humanities, Faculty of Medicine, University of Geneva, Geneva, Switzerland; 2 Philosophy Department, University of Lausanne, Lausanne, Switzerland; 3 Department of Clinical Neurosciences, Division of Neurorehabilitation, University Hospitals of Geneva, Geneva, Switzerland

**Keywords:** citizen science, materiovigilance, research ethics, safety, transcranial brain stimulation

## Abstract

Transcranial direct current stimulation (tDCS) is a non-invasive neuromodulation technique that delivers weak electrical currents to targeted brain regions in order to modulate neural activity and potentially enhance cognitive and motor functions. Clinical studies have reported benefits for conditions such as depression, stroke, and Parkinson's disease, typically with only minor and transient side effects. While short-term safety is relatively well established, the long-term effects require more systematic data and therefore require caution. At the same time, tDCS is increasingly being used at home without supervision for extended periods, as devices can be purchased online without prescription or even self-assembled. This situation highlights the need for appropriate safeguards to protect unsupervised users and to ensure that research into supervised long-term use is conducted under ethically acceptable conditions. In this paper, we specifically assess the risks that may arise from prolonged and unsupervised application of tDCS and propose measures that should be implemented by researchers, regulators, and commercial stakeholders involved in the distribution of tDCS devices.

## 1 Introduction

Transcranial Direct-Current Stimulation (tDCS) is a technique that modulates the excitability of brain areas with electric currents in order to enhance functions such as memory, cognitive processing, and motor control (Day et al., 2023)^1^. It has the advantage of being non-invasive and relatively safe as it is usually painless and with little and minor side effects. The effects of this technique on different functions are under study. Benefits have been found for conditions such as depression, stroke, and Parkinson's disease. The side effects of short-term use and safety parameters are well described (Antal et al., 2025). Numerous trials have shown that using the technique at home under supervision is also safe and that few major adverse events are reported (Antal et al., 2025; Shaw et al., 2017; Sandran et al., 2019; Rossi et al., 2021).

However, as most trials take place over a few weeks (often 30 days) the effects of long-term use (from 30 days to months or years) still require more systematic data. While long-term supervised use does not appear dangerous, more research to identify both risks and benefits is needed, especially given that most of the time, only the effects on the targeted conditions are monitored.

Precautions are especially needed given that in recent years, companies have started selling devices to individuals without prescription. The fact that the technique can be easily accessed and used regularly, and long-term, without supervision (Wurzman et al., 2016) raises several ethical issues that have so far been overlooked.

In this paper, we discuss how we should address these issues from a clinical and ethical perspective. We discuss what we can expect in terms of risks of long-term use and recommend measures to be introduced in supervised long-term trials to ensure that the collection of data regarding the effects of long-term use of tDCS is ethically acceptable. We also define the conditions that must be met to sell tDCS devices to individuals outside of a supervised setting.

## 2 Effects and risks of tDCS

Transcranial direct current stimulation (tDCS) is a non-invasive brain stimulation technique that modulates neuronal activity by delivering a weak, constant electrical current through electrodes placed on the scalp (Nitsche and Paulus, 2011; Wurzman et al., 2016). It is thought that, depending on the polarity of the applied stimulation, tDCS can either enhance (anodal stimulation) or inhibit (cathodal stimulation) cortical excitability.

Initially developed for research purposes, tDCS has gained attention in clinical and therapeutic settings for its potential to improve motor, cognitive, and psychiatric functions (Chase et al., 2020; Lefaucheur et al., 2017). It has been studied for various applications, including post-stroke motor rehabilitation, treatment of depression, and enhancement of learning and memory.

tDCS impacts neural function at multiple levels, including synaptic plasticity, neurotransmitter, neural oscillations and network interactions (Das et al., 2016; Stagg et al., 2018). Many of these complex effects are only partially understood, even in the short term.

Current tDCS protocols use current intensities that are over an order of magnitude below those that can induce brain injury. When tDCS is applied according to current safety recommendations ( ≤ 40 min, ≤ 4 milliamperes, ≤ 7.2 Coulombs) no serious adverse effect or irreversible injury has been reported (Antal et al., 2017, 2025; Bikson et al., 2016). The most common mild and reversible adverse effects are skin redness and an itching or burning sensation (Antal et al., 2025).

## 3 Risk of long-term use and of multiple sessions a day

Most clinical trials of tDCS last up to 30 days. Since home-based clinical trials have been found to be possible and safe (Charvet et al., 2020), more long trials have been conducted. In Borrione et al. (2024) conducted a large-scale randomized-controlled trial over 6 weeks in which one of the three arms received stimulation every week day for 3 weeks and then twice-weekly for another 3 weeks. Another recent large-scale randomized-controlled trial has demonstrated that longer-term home use (10 weeks) of tDCS can be beneficial for treating depression (Woodham et al., 2025). Pilloni et al. (2022) analyzed data from several trials of 308 participants who underwent 10 to 60 daily applications (with an average of 23 sessions). In 2019, a study found that using tDCS daily over 6 months had positive effects on Alzheimer's disease (Im et al., 2019), though only 11 patients were in the active group. Similarly, Madhavan et al. (2025) conducted a trial in which one experimental arm was administered three sessions a week for 24 weeks, though only seven participants were in that arm. In most studies, tDCS was well tolerated and no adverse event was reported. However, Kumpf et al. (2023) reported having to interrupt a trial because of burns. A risk of mania or hypomania has also been observed (Kang et al., 2024). This suggests that caution is important when teaching tDCS to participants or carers, and that appropriate monitoring is necessary. Furthermore, more systematic data are needed (Antal et al., 2025).

The effects of multiple sessions a day have been less studied than the ones of long-term use, although several studies have been published in the past 7 years. No study reported serious adverse events, and the multiple sessions were well tolerated. Several studies administered two sessions a day for 5 days (Jafari et al., 2021; Aksu et al., 2022; Moirand et al., 2022). Other studies tested two sessions a day for 10 days (Bation et al., 2019; Zanardi et al., 2020; Hsu et al., 2023) and 12 days (Pinto et al., 2021). Lastly, Couture et al. (2025) administered five sessions a day over 10 days. While they reported no serious adverse effects, “64.3% of participants presented with mild irritative contact dermatitis” (Couture et al., 2025) which they suspect is “related to current directionality and the inherent intensive aspect of [their] protocol” (Miron et al., 2023). Hence, while the results are reassuring, they remain limited and more systematic data is required (Antal et al., 2025).

## 4 Potential risks related to meta- and competitive plasticity

In general, it is expected that repetitive exposure will produce similar effects in each session, such that the effects observed in a single session can be enhanced and stabilized over time. However, principles of plasticity suggest that this may not be the case. At least in theory, there are some risks inherent to repetitive exposure that could reduce or even paradoxically invert the effects.

One critical aspect is the phenomenon of meta-plasticity—the brain's ability to regulate its own plasticity. This regulatory mechanism, while fundamental to adaptive learning and neural recovery, might paradoxically pose risks when chronically modulated by external stimulation. Meta-plasticity describes how prior neural activity or interventions influence the subsequent plastic potential of neuronal circuits. The state-dependency of neuromodulation, as highlighted by Silvanto, Muggleton, and Walsh for Transcranial Magnetic Stimulation (TMS) (Silvanto and Pascual-Leone, 2008), underscores that the effects of brain stimulation are contingent upon the pre-existing functional state of the targeted neurons. Repeated exposure to neuromodulation techniques like TMS might not only alter but also potentially reverse the intended effects under certain conditions (Silvanto and Pascual-Leone, 2008). It is possible that similar effects could be found with tDCS. For instance, preconditioning tDCS has been shown to modify and even reverse the effects of subsequent repetitive transcranial magnetic stimulation (rTMS) on motor cortical excitability (Cosentino et al., 2012; Siebner et al., 2004), illustrating the potential complexity of multiple neuromodulation sessions. This reversal occurs due to homeostatic plasticity mechanisms, where the brain compensates for repeated stimulation by decreasing, rather than increasing, synaptic efficacy.

In the context of long-term tDCS use, it is possible that such homeostatic responses could manifest as diminished therapeutic efficacy or even maladaptive plasticity. For instance, chronic stimulation targeting motor or cognitive networks might lead to reduced responsiveness over time, undermining the intended benefits of sustained treatment. This hypothesis has not been tested so far and further research is needed.

Another critical consideration is that enhancing certain brain areas and functions through tDCS may possibly lead to compensatory inhibition of others. This phenomenon, often referred to as “competitive plasticity” (Medini, 2014), highlights the interconnected nature of neural networks. When one region is stimulated to enhance specific functions, it can disrupt the balance of activity across the network, potentially suppressing adjacent or functionally related regions (Mackwood et al., 2021). There is some evidence that this can be the case with tDCS. For example, Iuculano and Kadosh, 2013 demonstrated that six consecutive days of tDCS to the posterior parietal cortex facilitated numerical learning but impaired automaticity for the learned material. In contrast, stimulation to the dorsolateral prefrontal cortex improved automaticity but impaired numerical learning. However, since most studies only investigate the effect of tDCS on the targeted function and do not examine other cognitive or affective functions, further research is required.

If these compensatory effects are proved to happen, they would be particularly concerning in clinical populations, where imbalances in neural activity may exacerbate existing deficits. Prolonged stimulation might unintentionally reinforce maladaptive patterns or reduce the capacity for spontaneous recovery in inhibited regions. Ethical considerations would thus extend to carefully evaluating the potential trade-offs in function and monitoring unintended consequences over time.

It is important to note, however, that tDCS primarily targets excitability (i.e., function) of superficial cortical areas. As such, we do not expect significant effects on complex functions reliant on long-term structural changes in deeper brain circuits, such as those associated with the sense of identity or emotional regulation. Additionally, the effects of tDCS are likely to be reversible, particularly when tDCS is used within established safety guidelines (Antal et al., 2025).

## 5 Ethical considerations for gathering evidence of effects and side effects of long-term use

Given that tDCS devices can be built at home or ordered online without prescriptions, it is likely that some users apply the technique without supervision regularly and in the long term. Even if the risk of substantive and irreversible harm is low, there are some risks for users, some of which might be unknown. This raises ethical issues that have so far been overlooked. It is important to both minimize the risks and inform users so that they can make an autonomous decision about using the technique. In this section, we outline measures that should be implemented to study the effects of long-term use of tDCS with and without supervision, while ensuring safety and informed consent of users. We consider two main ways of gathering data on the risks of long-term use: clinical studies and materiovigilance.

### 5.1 Clinical studies

Clinical studies are critical for gathering reliable data on both benefits and side effects of long-term use of tDCS. While several trials of supervised long-term use of tDCS at home have been published, more systematic data is needed (Antal et al., 2025). For a study to be ethically acceptable, the expected risk-benefit balance must be assessed to be favorable overall. In the case of tDCS, the expected risk is low, and potential adverse events are likely reversible after the interruption of the treatment. Given that benefits of the technique have already been observed and are expected to be found as well in long-term use, the benefit-risk balance is most likely to be acceptable.

However, precautions should be taken. For instance, there should be regular monitoring and follow-up so that potential adverse effects can be identified, and treatment stopped as soon as possible if needed. Indeed, there are still studies that do not assess and report on adverse events (Kang et al., 2024) and most studies do not conduct baseline assessments, making it more difficult to determine if an adverse event is in fact a pre-existing symptom (Antal et al., 2025). Existing guidelines for trials of supervised use of tDCS at home should be scrupulously followed, including regarding the training for tDCS users (Charvet et al., 2020).

Furthermore, only participants suffering from a condition (such as depression for instance) should be recruited, while applications for cognitive enhancement are not justified while the risks of long-term use at home are not better known. An intervention is usually considered a therapy if it is used to treat a medical condition, while it is considered enhancement if it is used to improve performance, but not to relieve symptoms. While this distinction might appear straightforward, it is in fact difficult to find a criterion to distinguish between the two, and several competing accounts of this criterion have been proposed in the literature (Gyngell and Selgelid, 2016; Giubilini and Sanyal, 2015; Allhoff et al., 2010; Lavazza, 2019). Nevertheless, generally, therapy aims at relieving suffering or symptoms that disadvantage the patient compared to others, while enhancement aims at providing an advantage compared to others. It then follows that the benefits for people suffering from a condition that could be relieved by tDCS are clearer and could more easily justify taking some risks than for those who would use it for enhancement.

Informed consent is, as usual, very important. Participants should be informed that there is some residual uncertainty around the risks associated with certain applications of this technology, even if no substantive and irreversible risks are expected. They should also be screened for underlying conditions that could be associated with known side effects.

One difficulty with identifying both benefits and side effects is that the technique could impact several different functions and that some of those could fail to be identified if they are not purposely looked for. Currently, most studies only test the effects of tDCS on the targeted condition. We recommend that clinical studies include a wider panel of cognitive and affective tests. In addition, participants should also be advised to remain attentive to and report any cognitive and affective change, even if they appear unrelated to the treatment.

### 5.2 Materiovigilance

Data from unsupervised users cannot be reliably used to assess the benefits of the technique, but they can be useful to identify side effects. Unsupervised users should be warned about the risks of using the device and the residual uncertainty surrounding long-term use and asked, for example by the manufacturer of the device, to be attentive to any change in cognitive or affective function as well as other symptoms and to report them, even if they appear unrelated. Depending on the regulation of their country, reports could be made to the user's primary practitioner, to the local instance in charge of materiovigilance or to the manufacturer. Ideally, users should also be advised to track their use of tDCS so that in case of adverse effects, the data can be analyzed to assess if the effects were caused by the use of the device. Some devices are connected to apps via Internet of Things which can make it easy to collect data of use. Since those users will likely not perform systematic assessment of their cognitive and affective function like participants in clinical studies, they should also be advised to perform regularly a number of tests available online and to ask people close to them to tell them if they notice any change of which they might not be aware themselves. If possible, there should be a national register they could subscribe to so that they could be informed of proven risks reported through materiovigilance.

Encouraging the report of side effects, but also of benefits could not only enhance safety, but also provide relevant indications for future research and contribute to building knowledge in the field. Given that the technique has been popular among neuro- and biohackers that have been testing the technique both for their own benefits and to advance science, as a form of citizen science, such encouragement could have an impact (Wexler, 2017, 2018). Lastly, manufacturers should share anonymous data about usage and potential effects with the scientific community and ask users for consent when they buy a device.

Lastly, there is little knowledge of unsupervised use of tDCS. Although all the studies conducted on this population found that unsupervised users usually respect safety guidelines (Jwa, 2015; Wexler, 2017, 2018) more systematic data should be collected on this population.

Some of these measures are already required in countries in which tDCS devices are considered medical devices. Given that tDCS can be used for therapy, that it impacts physiology of the brain and that the risks of long-term use are not known yet, tDCS devices should, in our opinion, be considered medical devices, even if they are used for enhancement and not therapy (Maslen et al., 2014). The aim is not to over-regulate and restrict access to tDCS devices, but to make sure that there are safety controls and proper information for users (Antal et al., 2024).

## 6 Conclusion

In conclusion, even if the risk of harmful effects of long-term use of tDCS is low, some amount of caution is required. Since devices for tDCS are already available on the market without a prescription and can therefore be used regularly over a long time without supervision, it is important to gather more systematic data not only about the benefits of long-term use, but also about the risks. This should be done both via clinical studies and materiovigilance. Several measures need to be introduced to ensure informed consent of users and risk minimization in the process. In particular, clinical studies should include a wider panel of cognitive and affective tests. tDCS devices sold to individuals without prescription should be categorized as medical devices and buyers should be better informed and advised to perform tests to monitor side effects and to report any side effect observed.

## Data Availability

The original contributions presented in the study are included in the article/supplementary material, further inquiries can be directed to the corresponding author.

## Footnotes

1 tDCS is one of the techniques that involve transcranial electric stimulation (tES) along with transcranial random noise stimulation, transcranial temporal interference stimulation or transcutaneous spinal direct current stimulation. We have chosen to focus on tDCS because long-term interventions have so far been limited to tDCS and it is mostly tDCS devices that are available online and used at home unsupervised. However, the principles we discuss, and the conclusions can be extended to other tES techniques as well.
